# Hemodynamic alterations and their clinical implications in the vertebrobasilar system among patients with isolated posterior circulation ischemic vertigo

**DOI:** 10.3389/fneur.2024.1463042

**Published:** 2024-11-01

**Authors:** Xuhua Song, Jingwei Liang, Congzhe Tian

**Affiliations:** Department of Otorhinolaryngology, The Affiliated Hospital of Hebei University, Baoding, Hebei, China

**Keywords:** posterior circulation ischemic vertigo, vestibular peripheral vertigo, hemodynamic parameters, vascular ultrasound, diagnostic sensitivity and specificity

## Abstract

**Background:**

This research aimed to ascertain independent risk factors and the diagnostic value of vascular parameters in differentiating posterior circulation ischemic isolated vertigo (PCI-IV) from vestibular peripheral vertigo (VPV).

**Methods:**

This study involved 247 patients with acute-onset vertigo, categorized into two groups: PCI-IV and VPV. Multivariate logistic regression was conducted to pinpoint independent risk factors for PCI-IV.

**Results:**

The duration of vertigo, particularly episodes lasting more than a few hours, was a significant predictor of PCI-IV (OR = 2.183, *p* < 0.001). The presence of diabetes mellitus (OR = 1.746, *p* = 0.008) and hypertension (OR = 2.291, *p* = 0.004) also notably increased the likelihood of PCI-IV. Hemodynamic measurements such as the inner diameter and average blood flow velocity (Vmean) of the vertebral artery, as well as the resistive index (RI), were identified as significant predictive factors (*p* ≤ 0.033). The ROC analysis demonstrated the vertebral artery RI had the highest diagnostic accuracy with an area under the curve (AUC) of 0.78, indicating an optimal balance between sensitivity and specificity.

**Conclusion:**

Clinical features such as the duration of vertigo, diabetes mellitus, and hypertension, along with vascular hemodynamics, are crucial in assessing the risk of PCI-IV. The RI in the vertebral artery emerged as a particularly potent diagnostic parameter. These findings advocate a multifaceted diagnostic approach, combining clinical and vascular parameters for the effective identification and management of PCI-IV.

## Introduction

Vertigo, with its multifaceted clinical presentations and underlying causes, remains one of the most prevalent neurological conundrums, posing significant diagnostic challenges across various medical specialties including neurology, otolaryngology, and surgery (1–3). The condition is generally classified into vestibular or non-vestibular categories, with peripheral vestibular vertigo encompassing benign paroxysmal positional vertigo (BPPV), Meniere’s disease (MD), and vestibular neuritis (VN). Central vestibular vertigo is most commonly associated with posterior circulation ischemia (PCI), adding another layer of complexity to the differential diagnosis of vertigo (4, 5).

BPPV, which represents 20–30% of vestibular disorders, is characterized by transient but intense dizziness episodes caused by certain head movements (6, 7). This condition is often due to otolith particles becoming dislodged within the semicircular canals. MD is characterized by episodic vertigo, fluctuating hearing loss, and tinnitus. The condition’s prevalence ranges widely from 3.5 to 513 per 100,000 individuals and is more commonly diagnosed in older adults, particularly among white females (8, 9). The pathogenesis of MD is thought to be associated with endolymphatic hydrops (10). VN, often leading to “secondary” BPPV, accounts for approximately 7 to 17% of cases. It is characterized by an acute or subacute onset of persistent vertigo without accompanying hearing loss, typically following a viral infection. PCI, which includes transient ischemic attacks and cerebral infarctions affecting the posterior cerebral circulation, accounts for approximately 20% of all ischemic strokes (11). PCI tends to have more severe consequences than strokes in the anterior circulation, especially when critical areas such as the brainstem or cerebellum are involved, leading to increased morbidity and mortality (12). The diagnosis of PCI is challenging due to its sudden onset, severe symptoms, often minimal clinical signs, and typically varied duration from several minutes to even days and months. This complexity highlights the critical need for accurately distinguishing central causes of vertigo, like PCI, from peripheral ones, particularly in cases of isolated vertigo where specific neurological signs or symptoms may not be present.

Differentiating central vertigo, as seen in PCI, from peripheral vertigo syndromes presents considerable clinical challenges. The deployment of non-invasive and cost-effective diagnostic tools such as carotid artery ultrasound (CAU) and transcranial Doppler (TCD) imaging is vital due to their sensitive detection capabilities. This study employs TCD and CAU to examine the structural and hemodynamic differences within the vertebrobasilar system among patients with vertigo of peripheral and central origins. We endeavor to clarify the diagnostic value of vertebrobasilar hemodynamics for identifying posterior circulation ischemic isolated vertigo (PCI-IV) in comparison to peripheral vertigo, aiming to enhance clinical assessment and management approaches for this intricate condition.

## Methods

### Study design and patient selection

This study retrospectively analyzed 247 patients over 18 years of age who presented chief complaint with acute onset vertigo at our outpatient department. The cohort included 92 patients with posterior circulation ischemic isolated vertigo (PCI-IV) and 155 patients with vestibular peripheral vertigo (VPV) (Supplementary Figure S1). Patients were selected based on their primary complaint of vertigo, and the inclusion criteria were defined according to the diagnostic standards of the respective vertigo types. The study was approved by the ethics committee of the Affiliated Hospital of Hebei University (HDFYLL-KY-2023-156), and written informed consent was waived since this is a retrospective study.

### Inclusion criteria

Patients with BPPV met the international diagnostic criteria set by the Barany Society, which included typical episodic positional vertigo or dizziness induced by changes in head position, usually from sitting to lying down or when turning over in bed. The vertigo lasted less than one minute, with positive Dix-Hallpike or Roll tests, and no inner ear or neurological localization symptoms or signs attributable to other diseases. Criteria for MD include at least two spontaneous vertigo episodes lasting from 20 min to 12 h and audiometric confirmation of low- to mid-frequency sensorineural hearing loss on the affected side, along with fluctuating aural fullness, tinnitus, or hearing loss, excluding other vertigo causes. VN is indicated by acute onset, vertigo lasting more than 24 h but less than a week, continuous direction-fixed spontaneous nystagmus, and post-vertigo imbalance lasting from days to weeks, without hearing loss or neurological symptoms. PCI-IV is diagnosed based on the Chinese consensus on posterior circulation ischemia, including transient ischemic attack (TIA) symptoms and vertigo linked to posterior circulation stroke, with or without autonomic dysfunction.

Patients received a brain MRI and TCD assessment. Any detected alterations in blood flow velocity or arterial narrowing prompted further examination with magnetic resonance angiography (MRA) or computed tomography angiography (CTA) of the head and neck. MRI scans were conducted on a GE Signa 3.0 T superconducting system with a 5 mm slice thickness and a 1.5 mm gap for plain scans, and a 6 mm thickness with a 1.2 mm interval for vascular imaging. Head and neck CTA utilized a Philips 64-slice spiral CT for three-dimensional reconstructions.

### Exclusion criteria

Individuals with a history of neck trauma, auditory or visual deficits; those diagnosed with cervical, cardiac, or ocular vertigo; and non-vestibular vertigo cases were excluded from the study. Additionally, patients with confirmed cardiovascular and cerebrovascular diseases or those who declined TCD or CAU testing were not included in the research.

### Carotid artery ultrasound (CAU) and transcranial Doppler (TCD) examinations

Both TCD and CAU examinations were performed by experienced ultrasound specialists. All TCD examinations were conducted by one expert, while all CAU examinations were conducted by another expert. CAU evaluations were conducted following a 30-min rest period and after discontinuation of any vasoactive medications. Utilizing a Toshiba Aplio500 color Doppler ultrasound system with an 11 L4 linear array probe (frequency range 5–12 MHz), patients were assessed while supine, with their necks exposed. The procedure involved two-dimensional grayscale ultrasound to examine vessel paths and lumen conditions for any signs of stenosis, dilation, or tortuosity. Measurements of the vertebral artery inner diameter were taken between the C2 and C6 vertebrae levels. The sampling volume was adjusted to one-third of the artery’s diameter, angled at 60° to the direction of blood flow, and the mean of three consistent readings was recorded.

TCD examinations used a German DWL-BOXTCD device with a 2 MHz probe. In a lateral position, the probe’s location and angle were optimized along with detector depth, transmission power, gain, and sampling volume for clarity. The assessment included bilateral vertebral arteries and the basilar artery, focusing on the average blood flow velocity (Vmean), resistive index (RI), and pulsatility index (PI), and again, the average of three measurements was considered for accuracy.

### Data collection

Baseline patient information was collected, including age, gender, smoking status, and medical history such as diabetes, hyperlipidemia, hypertension, and history of vertigo. Detailed information regarding the onset of vertigo, its association with positional changes, duration, and primary clinical manifestations such as nausea, vomiting, and tinnitus were also recorded.

### Statistical analysis

We compared the means of continuous variables using the independent samples t-test and analyzed categorical data using the Chi-square test or Fisher’s exact test where appropriate. The significance of hemodynamic parameters in diagnosing PCI-IV was evaluated using Receiver Operating Characteristic (ROC) curve analysis. Multivariate logistic regression was conducted to identify independent risk factors for PCI-IV, including clinical and vascular parameters. Data were analyzed using SPSS version 20 (SPSS, Chicago, IL), with a *p*-value of less than 0.05 considered statistically significant.

## Results

### Demographic and clinical characteristics

We assessed the demographic and clinical features of 247 patients who presented with acute-onset vertigo in our department (Table 1), all of whom were over 18 years of age. The cohort was categorized into two groups: those diagnosed with PCI-IV (*n* = 92) and those with VPV (*n* = 155). The VPV group included patients with BPPV (*n* = 96), MD (*n* = 31), and VN (*n* = 28). The PCI-IV group was comprised of individuals with cerebral infarction in the posterior circulation blood supply area (*n* = 74) and transient ischemic attack (*n* = 18). The mean age was 54.18 ± 9.63 years for the VPV group and 56.57 ± 11.04 years for the PCI-IV group, with no significant statistical difference (*p* = 0.351). Gender distribution across the groups was also statistically comparable, with 49.7% males in the VPV group and 57.6% in the PCI-IV group (*p* = 0.238). Notable clinical differences were identified between the groups. Duration of vertigo was significantly different; a larger proportion of the PCI-IV group experienced vertigo for a few hours compared to the VPV group (52.2% vs. 17.4%, *p* < 0.001). The incidence of diabetes mellitus was higher in the PCI-IV group (33.7% vs. 20.6%, *p* = 0.034), as was hypertension (45.7% vs. 26.5%, *p* = 0.008). The proportion of patients with hyperlipidemia was greater in the PCI-IV group compared to the VPV group, although this did not reach statistical significance (41.3% vs. 29.0%, *p* = 0.052). Furthermore, patients with PCI-IV were more likely to have a history of vertigo lasting for more than six months (14.1% vs. 5.2%, *p* = 0.019). Other clinical manifestations such as nausea and vomiting, tinnitus, and nystagmus did not show significant differences between the two groups (*p* = 0.229, *p* = 0.061, and *p* = 0.754, respectively).

**Table 1:** Demographic and clinical characteristics of patients with posterior circulation ischemic isolated vertigo (PCI-IV) and vestibular peripheral vertigo (VPV).

Characteristics	VPV (*n* = 155)	PCI-IV (*n* = 92)	*p* value
Age (years)	54.18 ± 9.63	56.57 ± 11.04	0.351
Gender			
Male	77 (49.7%)	53 (57.6%)	0.238
Female	78 (50.3%)	39 (42.4%)	
Diagnosis			
Cerebral infarction in the posterior circulation blood supply area	-	74 (80.4%)	-
Transient ischemic attack	-	18 (19.6%)	
Benign paroxysmal positional vertigo	96 (61.9%)	-	
Meniere’s disease	31 (20%)	-	
Vestibular neuritis	28 (18.1%)	-	
Vertigo lasting time			
A few seconds	52 (33.5%)	5 (5.4%)	< 0.001
A few minutes	69 (44.5%)	36 (39.1%)	
A few hours	27 (17.4%)	48 (52.2%)	
A few days	7 (4.5%)	3 (3.3%)	
Diabetes mellitus			
Yes	32 (20.6%)	31 (33.7%)	0.034
No	123 (79.4%)	61 (66.3%)	
Hypertension			
Yes	41 (26.5%)	42 (45.7%)	0.008
No	114 (73.5%)	50 (54.3%)	
Hyperlipemia			
Yes	45 (29.0%)	38 (41.3%)	0.052
No	110 (71.0%)	54 (58.7%)	
More than six months of vertigo			
Yes	8 (5.2%)	13 (14.1%)	0.019
No	147 (94.8%)	79 (85.9%)	
Nausea and vomiting			
Yes	112 (72.3%)	73 (79.3%)	0.229
No	43 (27.7%)	19 (20.7%)	
Tinnitus			
Yes	29 (18.7%)	27 (29.3%)	0.061
No	126 (81.3%)	65 (70.7%)	
Nystagmus			
Yes	34 (21.9%)	22 (23.9%)	0.754
No	121 (78.1%)	70 (76.1%)	

The data were presented as mean ± SD or *n* (percentage). The comparisons of data were done by Mann–Whitney test or Fisher’s exact test or Chi-square test.

The data suggest that while age and gender did not differ significantly between patients with PCI-IV and VPV, there were significant differences in the duration of vertigo symptoms and the prevalence of comorbid conditions such as diabetes mellitus and hypertension. These findings highlight the importance of considering underlying metabolic and cardiovascular conditions in patients presenting with vertigo.

### Hemodynamic parameters in posterior circulation ischemic vertigo

**Figure 1:**
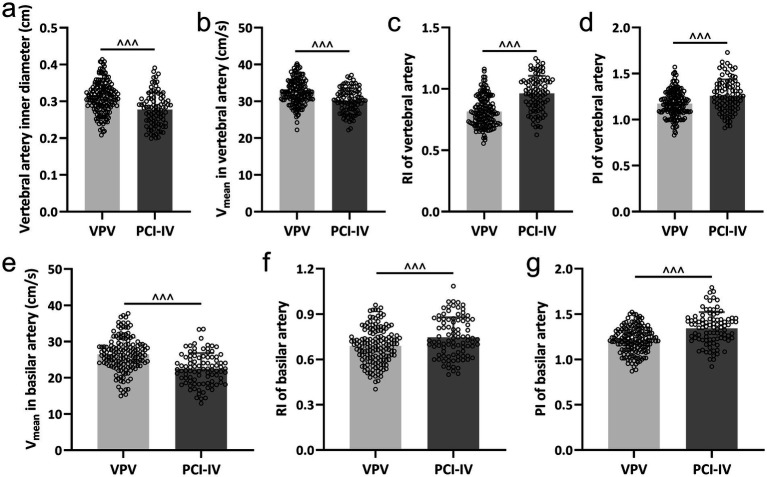
Comparative analysis of hemodynamic parameters in vertebrobasilar arteries. The differences between patients diagnosed with posterior circulation ischemic isolated vertigo (PCI-IV, *n* = 92) and those with vestibular peripheral vertigo (VPV, *n* = 155) in terms of the inner diameter of the vertebral artery (a), the mean blood flow velocity (Vmean) in the vertebral artery (b), the resistive index (RI) in the vertebral artery (c), the pulsatility index (PI) in the vertebral artery (d), the Vmean in the basilar artery (e), the RI in the basilar artery (f), and the PI in the basilar artery (g). Data are presented as mean ± standard deviation (SD). Statistical significance was determined using the Mann–Whitney test, with ^^^ indicating *p* < 0.001.

Our investigation identified significant hemodynamic discrepancies between the PCI-IV cohort and the VPV group. These variations were assessed through measurements of the vertebral and basilar arteries’ inner diameters, Vmean, RI, and PI. The inner diameter of the vertebral artery was considerably narrower in the PCI-IV group (Figure 1a). Additionally, the mean blood flow velocity was observed to be lower in the vertebral (Figure 1b) and basilar arteries (Figure 1e) among patients with PCI-IV compared to those with VPV. The resistive index was markedly elevated in both the vertebral (Figure 1c) and basilar arteries (Figure 1f) for the PCI-IV group, which indicates an increased resistance within the vascular system. Similarly, the pulsatility index was also higher in the PCI-IV group for both vertebral (Figure 1d) and basilar arteries (Figure 1g), reflecting potential heightened resistance to pulsatile blood flow or a diminished compliance of the distal vessels.

### Diagnostic efficacy of vascular parameters for posterior circulation ischemic vertigo

**Figure 2:**
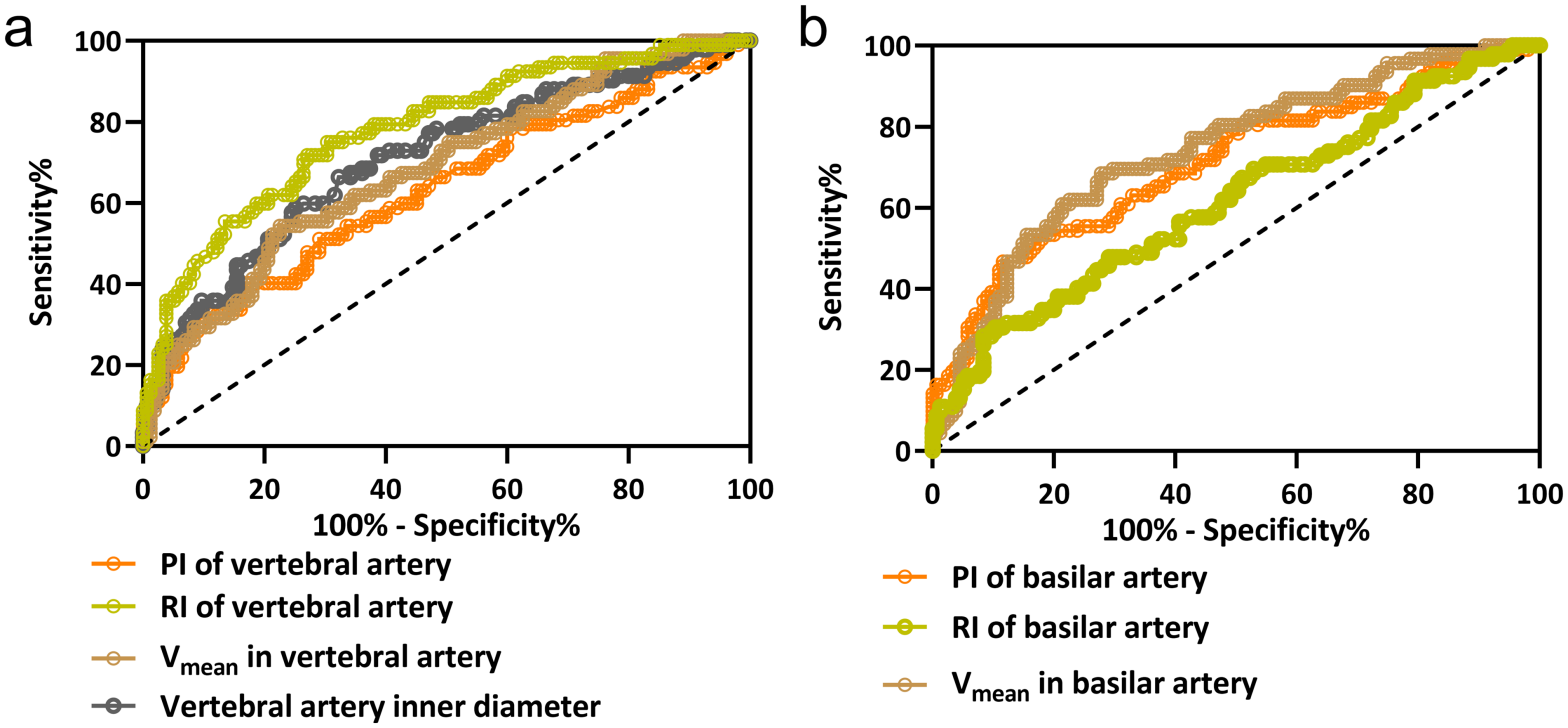
ROC curve analysis for hemodynamic parameters in vertebrobasilar vertigo diagnosis. The ROC curves for the vertebral artery inner diameter, mean blood flow velocity (Vmean), resistive index (RI), and pulsatility index (PI) in panel (a); and for the basilar artery’s Vmean, RI, and PI in panel (b). These curves evaluate the diagnostic performance of each parameter in distinguishing posterior circulation ischemic isolated vertigo (PCI-IV) from vestibular peripheral vertigo (VPV). Each curve is a graphical representation of the sensitivity versus 1-specificity for the respective hemodynamic parameter at various threshold settings.

The diagnostic capability of various vascular parameters to distinguish between PCI-IV and VPV was assessed using ROC analysis. The ROC curves, depicted in Figure 2, illustrate the sensitivity and specificity at different thresholds for the inner diameter, Vmean, RI, and PI of the vertebral artery (Figure 2a); and for the Vmean, RI, and PI of the basilar artery (Figure 2b). These curves elucidate each parameter efficiency in diagnosing PCI-IV. The vertebral artery RI demonstrated superior diagnostic accuracy with the highest area under the curve (AUC) of 0.78, suggesting an optimal balance of sensitivity (75%) and specificity (69.68%) at a threshold of 0.86. Similarly, the basilar artery’s Vmean displayed comparable efficacy with an AUC of 0.74, sensitivity of 68.48%, and specificity of 72.26% at the cut-off value of 24.09 cm/s. Comprehensive diagnostic metrics derived from the ROC analysis are detailed in Table 2. The Youden index, which quantifies the effectiveness of a diagnostic test, achieved its peak value for the RI of the vertebral artery, indicating its outstanding diagnostic value for PCI-IV among the parameters assessed. The distinction of AUC values from the line of no discrimination was statistically significant (*p* < 0.001 for most parameters), reaffirming the considerable potential of these vascular parameters to effectively discriminate between PCI-IV and VPV.

**Table 2:** Diagnostic values in ROC analysis.

	Cut-off value	AUC (95% CI)	*p*	Sensitivity (%)	Specificity (%)	Youden index
Vertebral artery						
Inner diameter	0.292 cm	0.71 (0.64 to 0.78)	<0.001	66.30	67.74	0.34
V_mean_	30.36 cm/s	0.68 (0.62 to 0.75)	<0.001	54.35	77.42	0.32
RI	0.86	0.78 (0.72 to 0.84)	<0.001	75.00	69.68	0.45
PI	1.30	0.63 (0.56 to 0.71)	<0.001	40.22	83.23	0.23
Basilar artery						
V_mean_	24.09 cm/s	0.74 (0.68 to 0.80)	<0.001	68.48	72.26	0.41
RI	0.84	0.62 (0.54 to 0.69)	0.002	30.43	89.68	0.20
PI	1.35	0.71 (0.64 to 0.78)	<0.001	53.26	81.94	0.35

CI, confidence interval.

### Independent risk factors for posterior circulation ischemic vertigo

A multivariate logistic regression analysis was conducted to identify the independent risk factors for PCI-IV in comparison to VPV. The variables from Table 1 and Figure 1 that exhibited significant differences were included as independent variables in this analysis, with the presence of PCI-IV as the dependent variable (PCI-IV = 1, VPV = 0). The duration of vertigo significantly influenced the likelihood of being diagnosed with PCI-IV, with a vertigo lasting time associated with more than a two-fold increase in the odds (OR = 2.183, 95% CI: 1.428 to 5.863, *p* < 0.001) (Table 3). Similarly, the presence of diabetes mellitus was associated with a substantial increase in the odds of PCI-IV (OR = 1.746, 95% CI: 1.259 to 4.225, *p* = 0.008). Hypertension was another significant predictor, more than doubling the odds of PCI-IV (OR = 2.291, 95% CI: 1.123 to 5.091, *p* = 0.004). An extended duration of vertigo, exceeding six months, also demonstrated a notable association with PCI-IV (OR = 1.635, 95% CI: 1.194 to 3.772, *p* = 0.017). In terms of vascular parameters, the vertebral artery inner diameter presented a strong association (OR = 1.834, 95% CI: 1.445 to 3.711, *p* = 0.014). The Vmean in the vertebral artery also emerged as a significant predictor (OR = 1.559, 95% CI: 1.062 to 2.869, *p* = 0.033). Furthermore, the RI of the vertebral artery was found to be a potent predictor, significantly increasing the odds of PCI-IV (OR = 2.337, 95% CI: 1.292 to 4.886, *p* = 0.003).

**Table 3:** Multivariate logistic analysis for posterior circulation ischemic isolated vertigo (PCI-IV) compared to vestibular peripheral vertigo (VPV).

	OR	95% CI	*p* value
Vertigo lasting time	2.183	1.428 to 5.863	< 0.001
Diabetes mellitus	1.746	1.259 to 4.225	0.008
Hypertension	2.291	1.123 to 5.091	0.004
More than six months of vertigo	1.635	1.194 to 3.772	0.017
Vertebral artery inner diameter	1.834	1.445 to 3.711	0.014
V_mean_ in vertebral artery	1.559	1.062 to 2.869	0.033
RI of vertebral artery	2.337	1.292 to 4.886	0.003
PI of vertebral artery	1.371	0.916 to 2.267	0.107
V_mean_ in basilar artery	1.904	1.338 to 4.572	0.005
RI of basilar artery	1.218	0.893 to 2.683	0.186
PI of basilar artery	1.384	1.075 to 3.192	0.039

OR, Odds Ratio; CI, confidence interval.

While the PI of the vertebral artery did not reach statistical significance (OR = 1.371, 95% CI: 0.916 to 2.267, *p* = 0.107), the Vmean in the basilar artery and the PI of the basilar artery were both significant predictors, with ORs of 1.904 (95% CI, 1.338 to 4.572, *p* = 0.005) and 1.384 (95% CI, 1.075 to 3.192, *p* = 0.039) respectively. The RI of the basilar artery did not demonstrate a significant association (OR = 1.218, 95% CI: 0.893 to 2.683, *p* = 0.186). These results underline the importance of a multifactorial approach in the assessment of PCI-IV, incorporating both clinical symptoms and vascular measurements to identify those at heightened risk.

## Discussion

Vertigo is a challenging clinical presentation with a spectrum of potential etiologies ranging from benign peripheral disorders to more serious central pathologies such as posterior circulation ischemic events (13). Accurate differentiation between PCI-IV and VPV is critical for appropriate management and prevention of potentially serious outcomes. In our comparative analysis, we investigated the demographic, clinical, and hemodynamic characteristics between patients with PCI-IV and VPV, as well as the diagnostic efficacy of various vascular parameters and the identification of independent risk factors for PCI-IV.

The demographic analysis of our patient cohort revealed no significant differences in mean age or gender distribution between the PCI-IV and VPV groups, which suggests that these demographic variables alone are not sufficient to distinguish between these conditions. The marked discrepancy in the duration of vertigo episodes between the two groups, wherein a larger proportion of PCI-IV patients experienced symptoms for a few hours, underscores the need for clinicians to regard the duration of vertigo as a potential indicator of underlying vascular events. This observation is in line with previous studies that suggest a transient ischemic attack or minor stroke in the posterior circulation could manifest as acute, short-lived vertigo (14, 15). The higher incidence of diabetes mellitus and hypertension in the PCI-IV group aligns with the established risk profile for cerebrovascular diseases (16, 17). These findings suggest that patients presenting with vertigo who also have these comorbid conditions should be carefully assessed for possible vascular etiologies, particularly in emergency settings where rapid differentiation is crucial for intervention. While not statistically significant, the trend toward a higher proportion of hyperlipidemia in the PCI-IV group may warrant attention. Hyperlipidemia is recognized as a modifiable risk factor for ischemic stroke (18), and its presence in patients with acute vertigo could inform the urgency and direction of diagnostic evaluations. The finding that patients with PCI-IV were more likely to report a history of vertigo lasting more than six months challenges the conventional perception of vertigo as a transient symptom in cerebrovascular disorders. It raises the question of whether recurrent or prolonged vertigo could be an early marker of progressive vascular impairment, warranting further investigation. The absence of significant differences in nausea, vomiting, tinnitus, and nystagmus between the groups highlights the nonspecific nature of these symptoms in diagnosing the etiology of vertigo. This reinforces the importance of comprehensive clinical evaluation over reliance on isolated symptomatology.

The hemodynamic parameters observed in our study elucidate underlying vascular disparities between patients with PCI-IV and those with VPV, which could have significant diagnostic and therapeutic implications. The narrowed inner diameter of the vertebral arteries observed in the PCI-IV group suggests vascular compromise that may predispose individuals to ischemic events. This finding is corroborated by the reduced mean blood flow velocity in the vertebral and basilar arteries, indicating a potential for ischemia in the posterior circulation. Our results resonate with the concept that vertebrobasilar insufficiency could present with vertigo symptoms (19, 20), and that these hemodynamic measures can serve as indicators for such pathology. The increased RI and PI in the PCI-IV group are indicative of increased resistance within the vascular system and reduced vessel compliance, respectively. The elevated RI and PI could reflect the systemic atherosclerotic burden or localized vascular pathologies, such as stenosis or small vessel disease (21, 22), which are known risk factors for stroke in the posterior circulation (23, 24). The hemodynamic differences may aid in differentiating between PCI-IV and VPV in acute settings, where rapid and accurate diagnosis is critical for the initiation of appropriate therapy. Our study suggests the inclusion of Doppler ultrasonography as a part of the routine evaluation for patients presenting with vertigo, particularly when there is a suspicion of ischemic etiology. The hemodynamic parameters measured in our study offer a window into the pathophysiological processes at play in PCI-IV. The data suggest that patients with PCI-IV may have underlying vascular conditions that not only predispose them to ischemic events but also to recurrent or chronic symptoms due to sustained hemodynamic impairment.

In addition, ROC analysis underscores the potential utility of these parameters in differentiating PCI-IV from VPV. The RI of the vertebral artery stands out with the highest AUC, suggesting an optimal balance of sensitivity and specificity. This aligns with the findings indicating that RI reflected the degree of downstream vascular resistance (25, 26), which is possibly altered in ischemic conditions affecting the vertebrobasilar territory (27). The Vmean of the basilar artery also demonstrates considerable diagnostic accuracy. These findings corroborate previous research suggesting that altered hemodynamics in the basilar artery are critical in the pathogenesis of posterior circulation events (28). The use of these parameters, particularly the RI and Vmean, in a clinical setting could enhance the diagnostic process for patients with vertigo, potentially leading to more timely and appropriate interventions. The multivariate logistic regression analysis elucidates several independent risk factors for PCI-IV. Notably, the duration of vertigo emerges as a significant predictor. This finding is particularly relevant, as it suggests that a longer duration of vertigo symptoms may be a red flag for a vascular rather than a vestibular etiology, an insight that could prompt earlier vascular imaging and intervention. Diabetes mellitus and hypertension being significant predictors reaffirms the association of these systemic conditions with cerebrovascular pathology (29–31). The strong association between these comorbidities and PCI-IV suggests that aggressive management of these conditions could be critical in the prevention of vertebrobasilar ischemic events. The identification of the vertebral artery inner diameter and Vmean as significant predictors further supports the role of vascular imaging in the assessment of vertigo. These parameters may serve as non-invasive markers for identifying patients at risk of PCI-IV, leading to earlier and more targeted treatment strategies.

Our results highlight the necessity of incorporating comprehensive diagnostic metrics, combining both clinical symptoms and vascular measurements, to improve the diagnostic accuracy for PCI-IV. The Youden index’s peak value for the RI of the vertebral artery indicates its potential as a standard screening tool in clinical practice. However, the PI of the vertebral artery’s lack of statistical significance suggests that it may have a limited role in isolation but could be considered in conjunction with other parameters.

## Conclusion

In conclusion, distinguishing between PCI-IV and VPV is complex but imperative for guiding treatment. The duration of vertigo, the presence of vascular risk factors, and specific hemodynamic parameters are key components in the evaluation of patients with vertigo. The identification of independent risk factors for PCI-IV supports a targeted approach to diagnosis and management, with implications for improving patient outcomes.

## Data Availability

The original contributions presented in the study are included in the article/Supplementary material, further inquiries can be directed to the corresponding author.
