# Supplementary figures and images for: Hemodynamic alterations and their clinical implications in the vertebrobasilar system among patients with isolated posterior circulation ischemic vertigo
Source: Front Neurol. 2024 Nov 1;15:1463042. doi: 10.3389/fneur.2024.1463042 (PMC11563943; doi:10.3389/fneur.2024.1463042)

## Supplementary materials

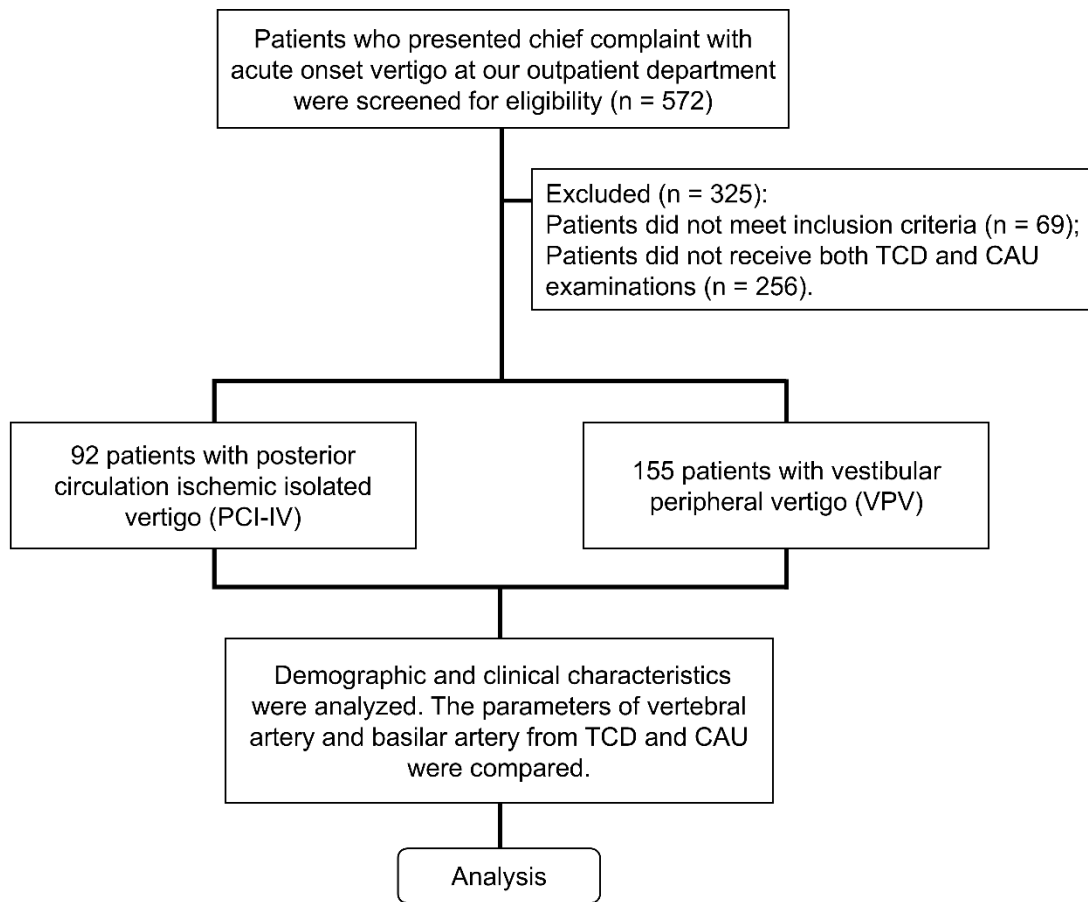

Figure S1. Chart flow of this study.

Supplement: Supplementary file 1 [file Data_Sheet_1.pdf]
